# Supplementary material for: The effect of conventional and sustainable agricultural management practices on carbon and water fluxes in a Mexican semi-arid region
Source: PeerJ. 2022 Dec 20;10:e14542. doi: 10.7717/peerj.14542 (PMC9784333; doi:10.7717/peerj.14542)
Supplement: Supplemental Information 1 [file peerj-10-14542-s001.pdf]

The effect of conventional and sustainable agricultural management practices on carbon and water fluxes in a Mexican semi-arid region

Gabriela Guillén-Cruz[1], Roberto Torres-Arreola [1], Zulia Mayari Sanchez-Mejia[2], Dulce Flores-Renteria[3]

[1] Sustentabilidad de los Recursos Naturales y Energía, Cinvestav Unidad Saltillo, Ramos Atizpe, Coahuila, Mexico

[2] Departamento de Ciencias del Agua y Medio Ambiente, Instituto Tecnologico de Sonora, Ciudad Obregon, Sonora, México

[3] CONACyT- Sustentabilidad de los Recursos Naturales y Energía, Cinvestav Unidad Saltillo, Ramos Atizpe, Coahuila, Mexico

Corresponding Author:

Dulce Flores-Renteria[3]
Av. Industria Metalúrgica 1062, Ramos Arizpe, Coahuila, 25900, México.
Email address: yaahid.flores@cinvestav.edu.mx.

Supplemental information

Figures: 4

Tables: 4

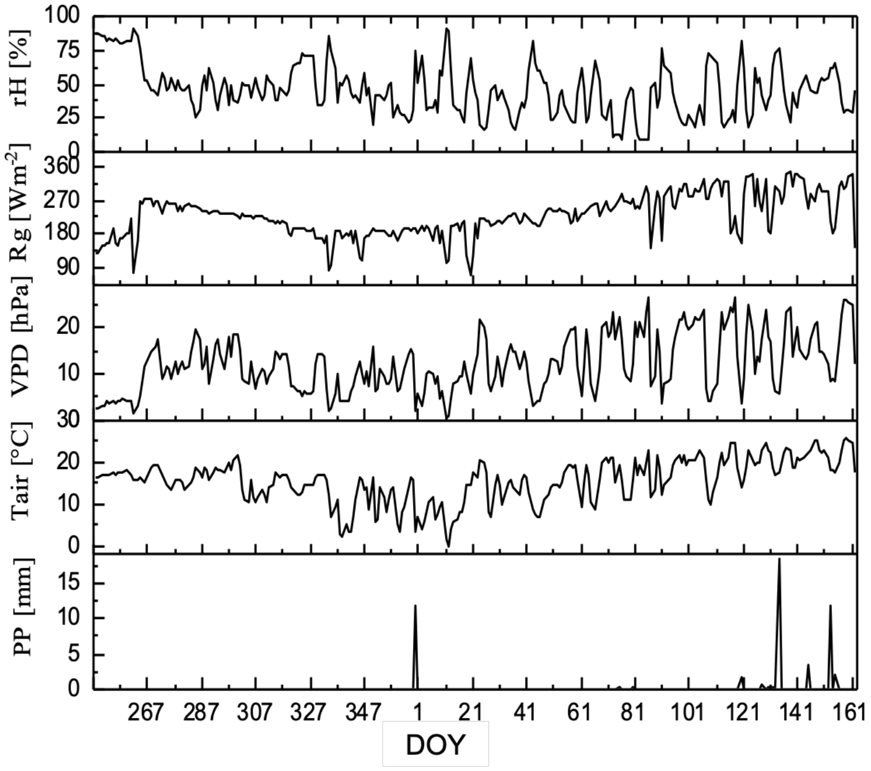

Figure S1. Environmental variation in the experimental plots of sustainable and conventional management in the Chihuahuan desert. Daily means of relative humidity (rH), global radiation (Rg), vapor pressure deficit (VPD), air temperature (Tair) and daily precipitation (PP).

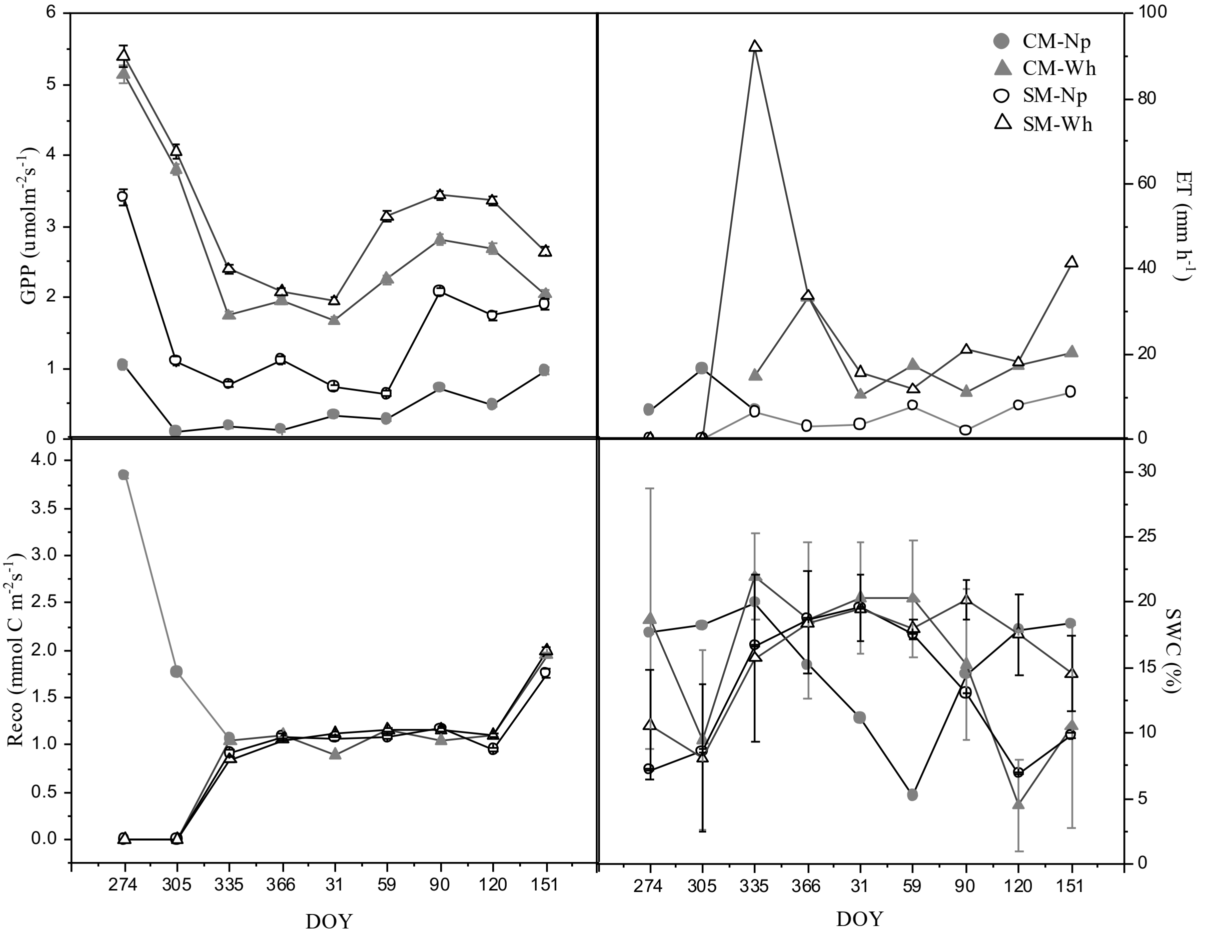

Figure S2. Monthly average of gross primary productivity (GPP), Evapotranspiration (ET), ecosystem respiration (Reco), soil water content (SWC), of nopal (Np) and wheat (Wh) crops subjected to conventional (CM) or sustainable (SM) management in the Chihuahuan desert.

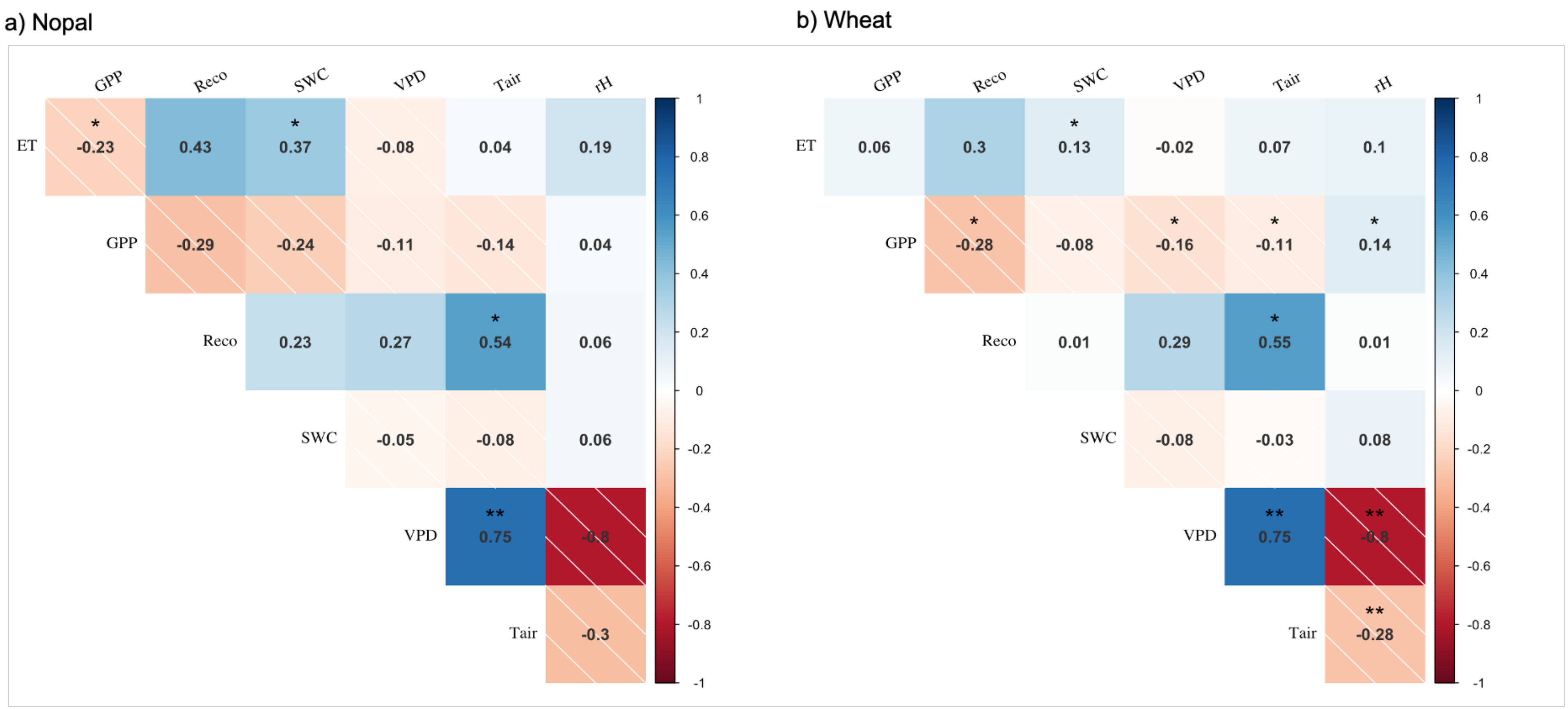

Figure S3. Correlation between environmental factors and carbon fluxes in the experimental plots. Significant Spearman's rho correlations are indicated by asterisks (significance level ***0.001, **0.01, *0.05). GPP= gross primary productivity, Reco= ecosystem respiration, SWC soil water content, VPD= vapor pressure deficit, Tair= air temperature. Daily mean data, n=636.

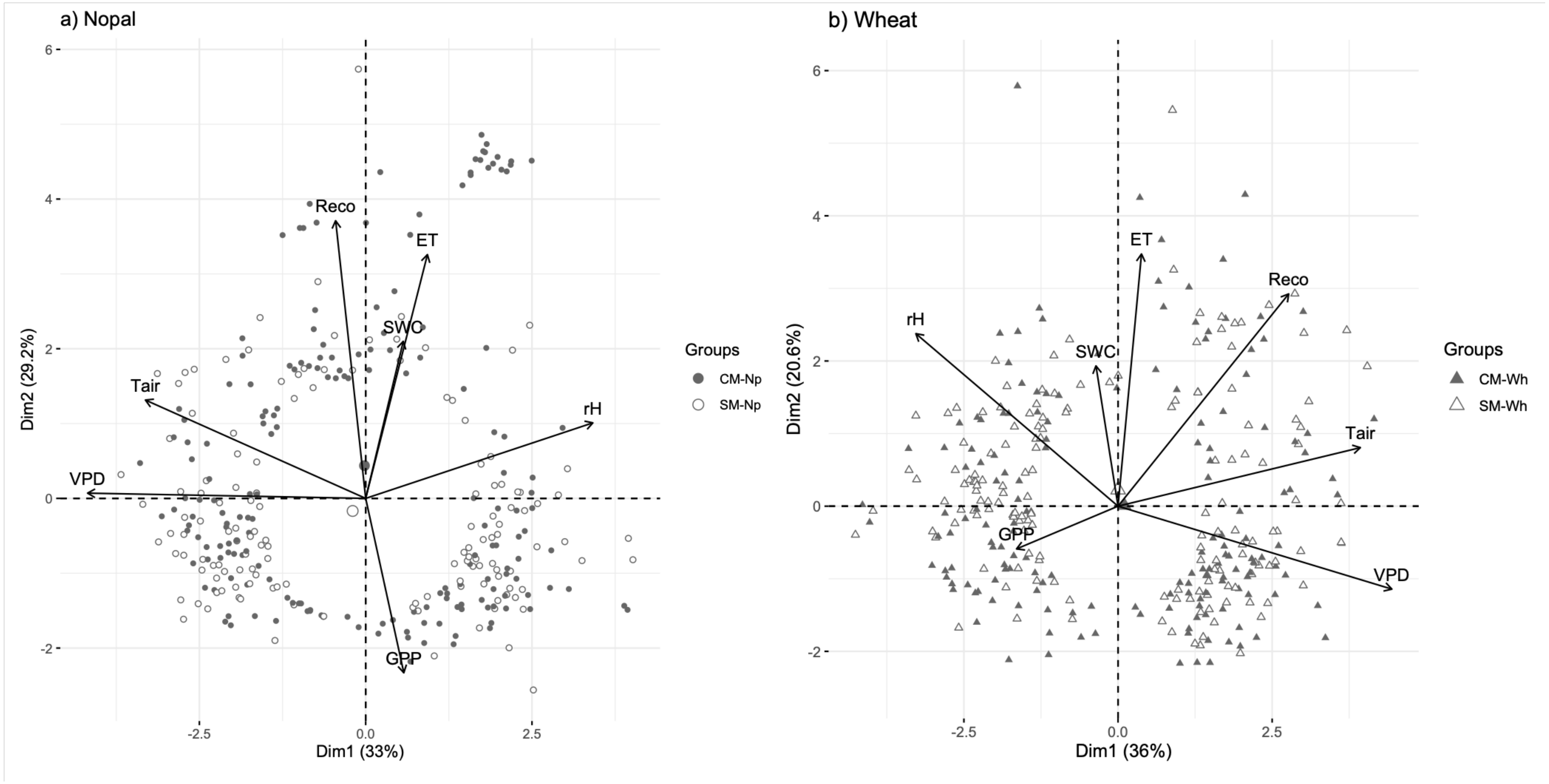

Figure S4. Principal Component Analysis for a) nopal (Np), and b) wheat (Wh) subjected to conventional (CM) or sustainable (SM) management in the Chihuahuan desert. GPP= gross primary productivity, Reco= ecosystem respiration, SWC soil water content, VPD= vapor pressure deficit, Tair= air temperature. Daily mean data, n=318.

Table S1. Analyses of variance for management effect (i.e., sustainable, or conventional) on nopal and wheat crops over evapotranspiration (ET), gross primary productivity (GPP), ecosystem respiration (Reco) and soil respiration (Rsoil). Significant effects are noted in bold.

		Nopal	Wheat
		**M**	**M**
**ET**	*F*	**4.99**	**6.85**
	p	**0.03**	**0.01**
	d.f.	231	401
**GPP**	*F*	17.95	2.74
	p	**<0.001**	0.10
	d.f.	231	401
**Reco**	*F*	159.38	1.06
	p	**<0.001**	0.30
	d.f.	231	401
**Rsoil**	*F*	1.44	0.37
	p	0.23	0.54
	d.f.	118	118

Table S2. Contribution percentage of each variable in the principal component analysis (first two principal components, PC2 and PC2).

	Contribution	
	PC1	PC2
Evapotranspiration (ET)	0.20	32.18
Gross primary productivity (GPP)	3.88	11.06
Ecosystem respiration (Reco)	8.23	31.45
Soil water content (SWC)	0.07	9.59
Vapor pressure deficit (VDP)	37.70	3.80
Air temperature (Tair)	29.01	0.35
Relative humidity (rH)	20.93	11.57
Nopal PCA		
Evapotranspiration (ET)	0.69	28.39
Gross primary productivity (GPP)	1.29	15.46
Ecosystem respiration (Reco)	1.58	35.02
Soil water content (SWC)	0.36	14.36
Vapor pressure deficit (VDP)	41.73	0.22
Air temperature (Tair)	28.15	2.40
Relative humidity (rH)	26.20	4.15
Wheat PCA		
Evapotranspiration (ET)	0.25	39.39
Gross primary productivity (GPP)	4.98	0.24
Ecosystem respiration (Reco)	14.05	25.91
Soil water content (SWC)	0.25	11.47
Vapor pressure deficit (VDP)	34.61	3.72
Air temperature (Tair)	26.79	2.25
Relative humidity (rH)	19.06	17.03

Table S3. Analyses of variance for management effect (i.e., sustainable, or conventional) on nopal and wheat crops over soil properties. Significant effects are noted in bold. d.f.= 22.

			Nopal	Wheat
			**M**	**M**
**Pos-tillage**	*pH*	*F*	2.18	**6.42**
		p	0.15	**0.02**
	Electric conductivity (mS cm^{-1})	*F*	0.21	**6.02**
		p	0.65	**0.02**
	Apparent density (g cm^{-3})	*F*	**17.99**	**6.83**
		p	**<0.001**	**0.02**
	Organic matter (%)	*F*	**32.35**	1.99
		p	**<0.001**	0.17
	Organic carbon (%)	*F*	**32.35**	7.33
		p	**<0.001**	0.02
	Available N (%)	*F*	0.21	0.26
		p	0.65	0.70
	Available P (ppm)	*F*	2.73	0.25
		p	0.11	0.59
**Pos-harvesting**	*pH*	*F*	0.09	**10.63**
		p	0.76	**<0.001**
	Electric conductivity (mS cm^{-1})	*F*	1.04	4.20
		p	0.32	0.05
	Bulk density (g cm^{-3})	*F*	0.29	**37.11**
		p	0.60	**<0.001**
	Organic matter (%)	*F*	0.04	**43.93**
		p	0.83	**<0.001**
	Organic carbon (%)	*F*	0.91	2.33
		p	0.35	0.14
	Available N (%)	*F*	2.18	**8.11**
		p	0.15	**0.01**
	Available P (ppm)	*F*	2.52	**10.28**
		p	0.13	**<0.001**

Table S4. Analyses of variance for management effect (i.e., sustainable, or conventional) on nopal and wheat crops over wheat properties. Significant effects are noted in bold. d.f.= 22.

		**Wheat**
		**M**
Total biomass (g)	*F*	**5.22**
	p	**0.03**
Stem lenght (cm)	*F*	2.52
	p	0.13
Root lenght (cm)	*F*	**19.94**
	p	**<0.001**
N (%)	*F*	**20.77**
	p	**<0.001**
P (%)	*F*	**82.64**
	p	**<0.001**
K (%)	*F*	**567.25**
	p	**<0.001**
Fe (ppm)	*F*	**18.87**
	p	**<0.001**
